# Optic nerve sheath enhancement in patients with a new diagnosis of idiopathic intracranial hypertension

**DOI:** 10.3389/fneur.2025.1707813

**Published:** 2025-12-01

**Authors:** Fernando Labella Álvarez, Amit M. Saindane, Valérie Biousse, Nancy J. Newman

**Affiliations:** 1 Department of Ophthalmology, Emory University School of Medicine, Atlanta, GA, United States; 2 Department of Morphological and Socio-Sanitary Sciences, Córdoba University School of Medicine and Nursing, Córdoba, Spain; 3 Department of Neurology, University Hospital Zürich, Zürich, Switzerland; 4 Department of Radiology and Imaging Sciences, Emory University School of Medicine, Atlanta, GA, United States; 5 Department of Neurological Surgery, Emory University School of Medicine, Atlanta, GA, United States; 6 Department of Neurology, Emory University School of Medicine, Atlanta, GA, United States

**Keywords:** idiopathic intracranial hypertension, optic nerve sheath enhancement, intracranial hypertension, misdiagnosis, optic perineuritis

## Abstract

**Introduction:**

Optic nerve sheath enhancement (ONSE) is a radiologic sign commonly associated with a diagnosis of optic perineuritis. However, recent studies have reported this radiologic sign in patients with idiopathic intracranial hypertension (IIH). The presence of ONSE in patients with IIH may lead to optic perineuritis diagnosis in excess. We evaluated the prevalence of ONSE in patients with a new diagnosis of IIH.

**Methods:**

Retrospective study of consecutive patients who presented to the emergency department of our quaternary care center with a suspected intracranial pressure disorder between June 15, 2023 and July 1, 2024. Patients who received a new diagnosis of IIH fulfilling the 2013 revised diagnostic criteria and who underwent orbital magnetic resonance imaging (MRI) with fat-suppressed contrast-enhanced sequences were included. An expert neuroradiologist evaluated all orbital MRIs for the presence of ONSE.

**Results:**

Forty-three patients with a new diagnosis of IIH were included (mean age, 31 ± 7 years; 100% women; 72% Black, 20% White, and 8% other). Of these 43 patients, three were categorized as having suggested ONSE versus blood vessels. The remaining 40 patients were categorized as having no ONSE.

**Discussion:**

ONSE is a radiologic sign that may occur in approximately 7% of patients with newly diagnosed IIH. However, the distinction between mild ONSE and blood vessels surrounding or within the optic nerve sheath is challenging. A location at the distal infraorbital portion of the optic nerve sheath and a corkscrew appearance are suggestive of a vascular etiology. Awareness of this radiologic sign in patients with IIH is important to avoid optic perineuritis diagnosis in excess.

## 1 Introduction

Optic nerve sheath enhancement is a known radiologic sign found in patients with inflammatory and infectious optic perineuritis, as well as with infiltrative meningeal processes (1) and giant cell arteritis (2).

A Chinese retrospective study (3), along with our recent case series (4), has reported the presence of optic nerve sheath enhancement in patients with primary and/or secondary causes of intracranial hypertension. In our experience, it is not uncommon that providers equate this radiologic finding with optic perineuritis, prompting unnecessary ancillary tests and treatment, increasing the risk of iatrogenesis.

The aim of our study was to evaluate the prevalence of optic nerve sheath enhancement in consecutive patients with newly diagnosed idiopathic intracranial hypertension (IIH). We hypothesized that optic nerve sheath enhancement may be a nonspecific radiologic sign of intracranial hypertension.

## 2 Materials and methods

This retrospective observational study evaluated all consecutive patients aged 18 years or older who presented to the emergency department of our quaternary care center between June 15, 2023, and July 1, 2024, with a suspected intracranial pressure disorder. Data were prospectively collected starting in June 2023 when we first implemented the routine use of a combined nonmydriatic ocular fundus photography and optical coherence tomography camera in our emergency department. An intracranial pressure disorder was suspected based on symptoms (headache, pulsatile tinnitus, and/or blurred vision) or signs (presumed papilledema on a prior fundus examination).

Patient medical records were reviewed. Patients with an ophthalmologic examination and available ocular fundus photography showing definite papilledema and who had received a new diagnosis of IIH meeting the 2013 revised diagnostic criteria (5) were included. Patients were excluded if orbital magnetic resonance imaging (MRI) with fat-suppressed contrast-enhanced sequences was unavailable, lumbar puncture was not performed within 48 h of MRI, lumbar puncture opening pressure was <25 cm of cerebrospinal fluid (CSF), or CSF findings were abnormal. Likewise, patients with an already known diagnosis of IIH, those with secondary causes of intracranial hypertension, or those with a clinical presentation suggestive of an alternative diagnosis were excluded. For included patients, data on sex, age, race, and body mass index (BMI) were collected from medical records. Overweight was defined as BMI 25–29.9 kg/m^2^ and obesity as BMI ≥30 kg/m^2^.

In all patients, MRIs were performed on 1.5-T or 3 T (Avanto or Trio, Siemens Healthcare) using a standard head coil. Orbital protocols included 3-mm coronal and axial T1-weighted, and 3-mm coronal and axial T2-weighted fat-saturated sequences, in addition to axial diffusion-weighted and T2-FLAIR images of the brain. Brain and orbit protocols included these same sequences with additional images of the brain. Intravenous gadolinium contrast was administered at a dose of 0.1 mmol/kg body weight, and postcontrast 3-mm coronal and axial T1-weighted fat-saturated images were acquired. One subspecialty-certified neuroradiologist (AS) reviewed all MRIs for the presence of optic nerve sheath enhancement in either eye.

Descriptive statistics with absolute and relative frequencies were used to summarize qualitative variables. Mean and SD (standard deviation) were reported for continuous variables. The Mann–Whitney *U* test was used for comparisons of quantitative variables, and the Fisher–Freeman–Halton exact test was applied for categorical variables. Statistical analysis was performed using IBM SPSS Statistics, version 31.

## 3 Results

**Figure 1:**
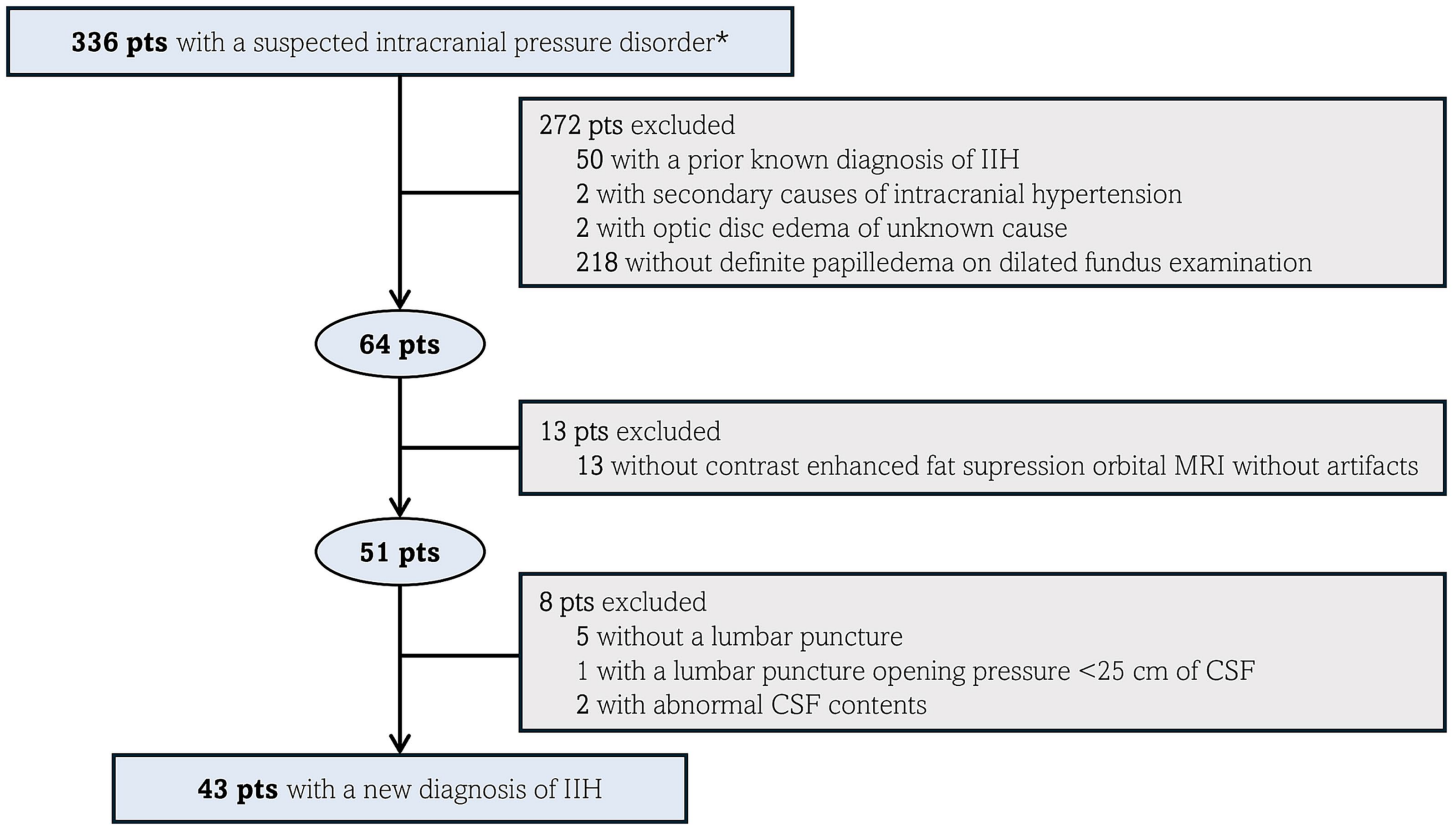
Exclusion criteria flow-chart in 336 patients with a suspected intracranial pressure disorder. ^*^Intracranial pressure disorder was suspected based on symptoms (headaches, pulsatile tinnitus and/or blurred vision) or signs (presumed papilledema on a prior fundus examination). CSF, cerebrospinal fluid; IIH, idiopathic intracranial hypertension; MRI, magnetic resonance imaging; pts, patients.

Of the 336 patients with suspected intracranial pressure disorder, 43 were included (Figure 1). The mean (±SD) age was 31 ± 7 years (range: 18–53). All 43 patients were female (100%). Of the 40 patients with known race, 29 (72%) were Black, eight (20%) were White, and three (8%) were categorized as Other. The mean (±SD) BMI was 37 ± 7 (range: 26–55). Thirty-seven patients (86%) were obese, and six (14%) were overweight.

**Figure 2:**
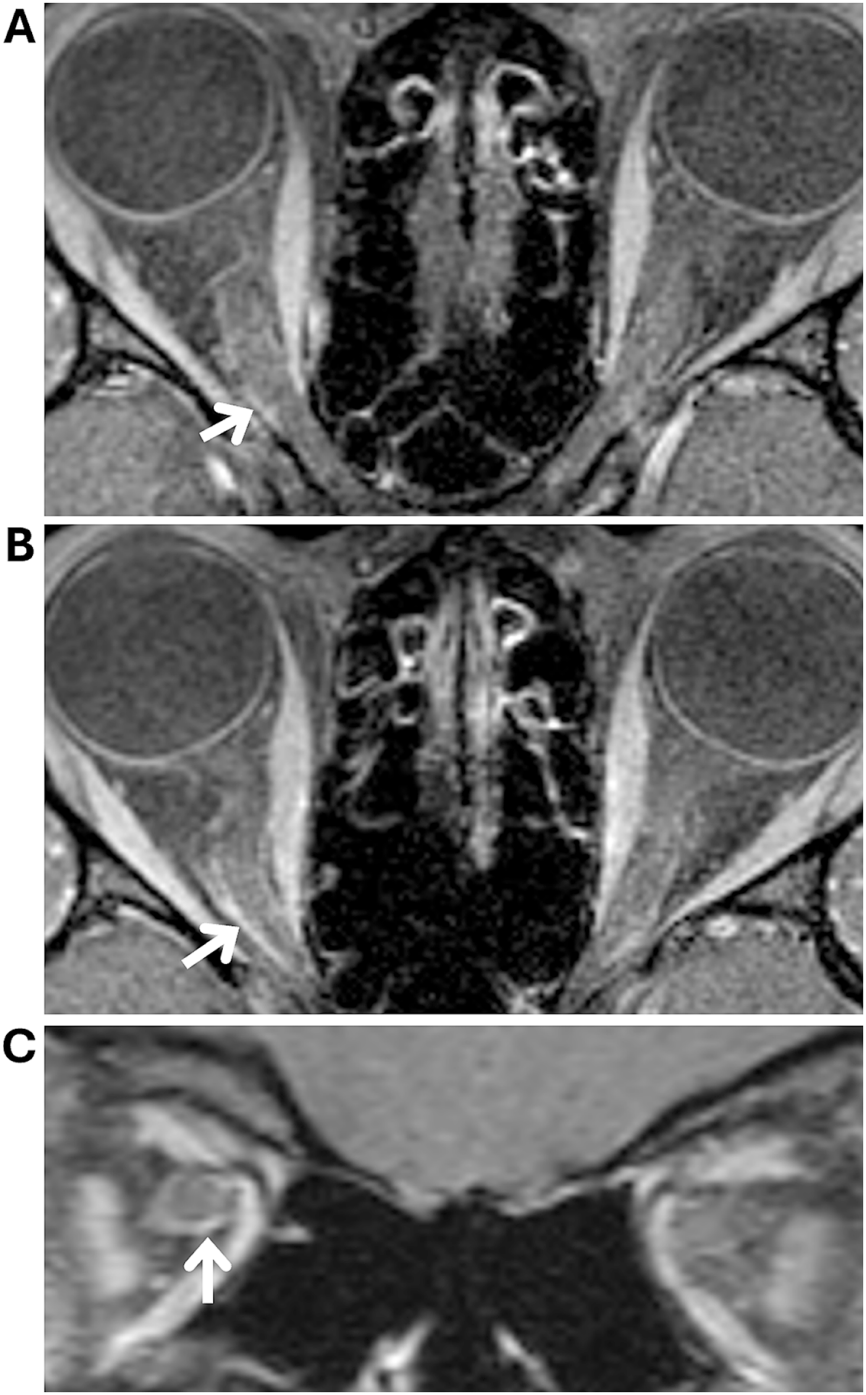
Magnetic resonance imaging (MRI) of a patient with a new diagnosis of idiopathic intracranial hypertension, categorized as having suggested optic nerve sheath enhancement versus blood vessels (patient two in Table 1). Fat-suppressed T1-weighted orbital MRI with contrast **(A–C)** in axial **(A,B)** and coronal **(C)** planes showing areas suggestive of optic nerve sheath enhancement (white arrows).

**Figure 3:**
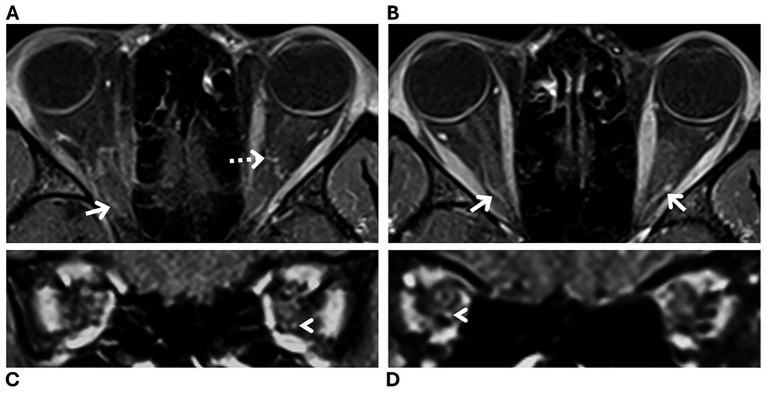
Magnetic resonance imaging (MRI) of a patient with a new diagnosis of idiopathic intracranial hypertension, categorized as having no definite optic nerve sheath enhancement. Fat-suppressed T1-weighted orbital MRI with contrast **(A–D)** in axial **(A,B)** and coronal **(C,D)** planes showing blood vessels in close relation to the optic nerve sheath complex (white arrows). The distal infraorbital location (arrowheads) and the corkscrew appearance (dashed arrow) are consistent with blood vessels rather than optic nerve sheath enhancement.

On MRI review, three patients (7%) were categorized as having suggested optic nerve sheath enhancement versus blood vessels in at least one eye (Figure 2), while the remaining 40 patients (93%) had no definite optic nerve sheath enhancement in either eye (Figure 3). Of the three patients with suggested optic nerve sheath enhancement, two (67%) were White, and one was Black (33%); among the 37 patients with known race and without enhancement, 28 (76%) were Black, 6 (16%) were White, and three (8%) were of other races (Fisher–Freeman–Halton exact test, *p* = 0.178). The mean (±SD) age was 30 ± 7 years (range, 22–36) in patients with suggested optic nerve sheath enhancement, and 31 ± 7 years (range, 18–53) in those without (Mann–Whitney *U*, *p* = 0.886). The mean (±SD) BMI was 39 ± 5 kg/m^2^ (range, 36–44) in patients with suggested optic nerve sheath enhancement and 36 ± 7 kg/m^2^ (range, 26–55) in those without (Mann–Whitney *U*, *p* = 0.431). On evaluation of radiologic signs of intracranial hypertension, all three patients with suggested optic nerve sheath enhancement had partially empty sella, transverse sinus stenosis, posterior globe flattening, enlarged Meckel’s caves, and optic nerve sheath distention. Two of these three patients also showed optic nerve tortuosity on MRI. Information regarding demographics, symptom onset, lumbar puncture and ophthalmologic examination of the three patients with suggested optic nerve sheath enhancement is summarized in Table 1.

**Table 1:** Three patients with newly diagnosed idiopathic intracranial hypertension and suggested optic nerve sheath enhancement on orbital magnetic resonance imaging.

Patient	Sex	Age (years)	Race	BMI (kg/m^2^)	Disease course^a^	Lumbar puncture		Ophthalmological examination				
						CSF content	OP (cm CSF)	Eye	Visual acuity^b^	Color vision^c^	Papilledema grade (mFS)	HVF 24-2 SITA fast description; MD (dB)
One	F	36	White	36	2 days	Normal	37	OD	20/20	14/14	Grade 1	Full; −1.41
								OS	20/20^−2^	14/14	Grade 1	Full; −1.14
Two	F	22	Black	35	Unknown^d^	Normal	37	OD	20/20	14/14	Grade 1	NSPD; −2.85
								OS	20/20	14/14	Grade 1	NSPD; −2.85
Three	F	31	White	44	Unknown^e^	Normal	36	OD	20/20^−1^	14/14	Grade 1	Full; 0.12
								OS	20/20	14/14	Grade 1	Full; 0.79

BMI, body mass index; CSF, cerebrospinal fluid; dB, decibels; F, female; HVF, Humphrey visual field; MD, mean deviation; mFS, modified Frisen scale; NSPD, nonspecific points of depression; OD, right eye; OP, opening pressure; OS, left eye; SITA, Swedish interactive thresholding algorithm.

a Disease course was defined as the time from symptom onset to diagnosis.

b Visual acuity was reported in Snellen notation.

c Color vision was assessed using Ishihara plates.

d Papilledema was suspected on a routine optometric examination in a patient with longstanding headache; worsening headaches and intracranial hypertension symptoms developed subsequently.

e Papilledema was suspected on a routine optometric examination; symptoms developed subsequently.

## 4 Discussion

**Figure 4:**
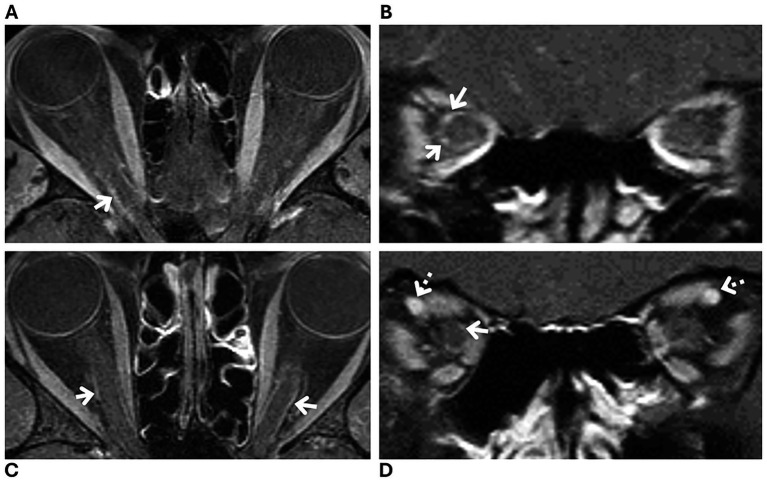
Magnetic resonance imaging (MRI) of two patients with a new diagnosis of idiopathic intracranial hypertension, showing prominent blood vessels within the optic nerve sheath (white arrows) without definite enhancement. Fat-suppressed T1-weighted orbital MRI with contrast **(A–D)** in axial **(A,C)** and coronal **(B,D)** planes: **(A,B)** from one patient and **(C,D)** from another. Blood vessels (white arrows) that mimic optic nerve sheath enhancement on axial planes **(A,C)** are more clearly identified as vessels on coronal planes **(B,D)**. Note the prominent ophthalmic vein on the coronal plane in one patient, suggestive of venous congestion (dashed arrows, **D**).

Optic nerve sheath enhancement has been recently described as a possible radiologic finding in patients with intracranial hypertension (3, 4). Our study showed the presence of possible optic nerve sheath enhancement in three of 43 (7%) patients with newly diagnosed IIH. In these three patients, optic nerve sheath enhancement was suggested with intermediate confidence by an expert neuroradiologist because blood vessels in close relationship to the optic nerve sheath could not be excluded as the anatomical underpinning of the radiologic finding. Although the distinction between optic nerve sheath enhancement and blood vessels can be challenging, a location at the distal infraorbital portion of the optic nerve sheath and a corkscrew appearance are both more suggestive of a vascular etiology (Figure 3). Likewise, orbital MRI on the coronal plane can help identify vessels within the optic nerve sheath (Figures 3, 4).

One retrospective study from China reported a higher frequency of optic nerve sheath enhancement in 82 patients diagnosed with IIH (15%) (3), compared with the 7% observed in our study. Their patients with optic nerve sheath enhancement had a longer disease course and a higher frequency of distension of the perioptic subarachnoid space (3). Whereas their study was conducted in an Asian population, most patients in our study were Black, and only one (3%) was Asian (3). Likewise, although our study population had a similar mean age, 31 versus 34 years, it had a higher percentage of females (100% versus 80%), and a higher percentage of patients with a BMI ≥28 (91% versus 46%), the cut-off value used in the Chinese study for obesity (3). In addition, compared with their patients with optic nerve sheath enhancement, who had optic nerve sheath distention (58%), bilateral transverse sinus stenosis (42%), and empty sella (67%), all three of our patients with suggested optic nerve sheath enhancement showed these radiologic signs of intracranial hypertension. Our three patients with suggested optic nerve sheath enhancement had mild papilledema and normal visual function in both eyes, except for nonspecific points of depression on HVF (Humphrey visual field) in one patient (patient two in Table 1). In contrast, the 12 patients with optic nerve sheath enhancement in the study from China (3) had a greater grade of papilledema, mean modified Frisen scale of 3.4, and worse visual function, with 11 of 24 eyes having moderate or severely decreased visual acuity (3). However, there was no statistically significant difference in visual function between their patients with and without optic nerve sheath enhancement (3). In our previous case series of four patients with intracranial hypertension and definite optic nerve sheath enhancement, three patients had secondary causes of intracranial hypertension and severe papilledema, while one patient was diagnosed with IIH and had mild papilledema (4). Similar to our study, all four patients had normal visual function except for mild changes on HVFs (4). This suggests that optic nerve enhancement may be present in patients with intracranial hypertension regardless of papilledema severity.

Although optic nerve sheath enhancement was associated with a longer disease course in patients with IIH in the Chinese study (3), establishing the disease onset in IIH patients is often challenging. Symptoms can be absent (6), not linked to disease onset, or confounded by a prior diagnosis of primary headache disorder (7, 8). In two of our three patients with suggested optic nerve sheath enhancement in this current study, papilledema was noted on a routine optometric examination, preceding either the onset of intracranial hypertension symptoms or the worsening of longstanding headache (patient two and three in Table 1). Therefore, disease onset could not be reliably determined in these patients. Of the 12 patients with IIH and optic nerve sheath enhancement in the study from China (3), four (33%) had been misdiagnosed with optic neuritis, ischemic optic neuropathy or another optic neuropathy. Since disease course was defined as the time from symptom onset to diagnosis, the longer course observed in patients with optic nerve sheath enhancement in their study may reflect the diagnostic delay related to a higher rate of misdiagnosis in this group. Recognizing suggested optic nerve sheath enhancement as a nonspecific radiologic sign of raised intracranial pressure may help avoid misdiagnosis and facilitate earlier diagnosis in these patients. Therefore, the presence of optic nerve sheath enhancement in patients with an otherwise typical presentation of IIH should not dissuade clinicians from making that diagnosis.

Optic perineuritis is an inflammatory process primarily involving the optic nerve sheath (1). It may occur as part of an orbital inflammatory disorder or in association with systemic inflammatory or infectious diseases (9). Whereas IIH typically presents with bilateral optic disc edema due to raised intracranial pressure, preserved visual function unless the optic disc edema is severe, and no pain with eye movements, optic perineuritis usually presents with unilateral, painful, subacute vision loss accompanied by optic disc edema (1, 9–11). Vision loss in optic perineuritis usually spares central vision (1, 9–11), however, concomitant optic nerve involvement, especially in cases secondary to myelin oligodendrocyte glycoprotein (MOG) antibody-associated disease (MOGAD) or sarcoidosis (12, 13), almost always causes decreased visual acuity and color vision. Alternatively, optic perineuritis can present as part of an orbital syndrome with proptosis, periocular edema, or limitation of eye movements (1). Although symptoms of raised intracranial pressure may help identify optic disc edema secondary to intracranial hypertension, optic perineuritis secondary to MOGAD (14), sarcoidosis (12), or syphilis (15–17), among other causes, can also be associated with elevated intracranial pressure and papilledema from meningeal involvement. Hence, symptoms and radiologic signs of intracranial hypertension should always be interpreted in context, and clinicians should look for other distinguishing features of optic perineuritis and optic neuritis, such as pain with eye movements and visual dysfunction. Patients with raised intracranial pressure and optic nerve sheath enhancement who do not fit the typical demographic for IIH of a young obese woman (18), should be evaluated for secondary causes of intracranial hypertension, while also considering the causes of optic perineuritis that can be associated with raised intracranial pressure (12, 16, 19). In addition, radiologic signs of orbital inflammatory disease, such as enhancement of the sclera, extraocular muscles, or streaky enhancement of the orbital fat (9–11), are more suggestive of optic perineuritis than the usually more subtle optic nerve sheath enhancement observed in our patients with IIH.

In the setting of raised intracranial pressure, venous stasis leading to orbital venous congestion and capillary leakage has been hypothesized as the underlying cause of true optic nerve sheath enhancement (3, 4). The occurrence of this radiologic sign in patients with intracranial hypertension may be determined by the degree of venous outflow blockade and the status of the extrajugular collateral pathways (20). Therefore, patients with more extensive optic nerve sheath venous collaterals and a relative lack of collateral venous pathways elsewhere would be more likely to develop optic nerve sheath enhancement. In our study, some patients without definite optic nerve sheath enhancement had prominent or enlarged orbital venous vasculature in close relation to the optic nerve and its sheath (Figure 4). It remains uncertain whether this radiologic finding, in the absence of associated optic nerve sheath enhancement, is nonspecific or reflects a milder spectrum of orbital venous congestion without capillary leakage in patients with intracranial hypertension. Evaluation of other signs of orbital venous engorgement in these patients might help clarify its clinical relevance.

Our study has several limitations. We did not perform repeat MRI in patients with suggested optic nerve sheath enhancement after papilledema resolution, which might have helped establish a causal relationship between intracranial hypertension and these optic nerve sheath findings. Likewise, the absence of a control group in our study prevents evaluation of the specificity of this radiologic finding. Indeed, a control group would help determine whether normal orbital vasculature in healthy subjects may also be misinterpreted as optic nerve sheath enhancement. In addition, although one strength of our study is that all patients underwent thin-section 1.5-T or 3-T fat-suppressed orbital MRI with contrast at the same facility, neither may be sufficiently sensitive to resolve small vessels within the optic nerve sheath. This is especially relevant at the orbital apex, where crowding of small blood vessels can be mistaken for optic nerve sheath enhancement, thereby overestimating its prevalence. Finally, we did not routinely test for anti-MOG antibodies. Although MOGAD has been associated with intracranial hypertension and optic nerve sheath enhancement (21), it rarely occurs as an isolated intracranial hypertension syndrome (19, 22, 23). Patients with MOGAD and raised intracranial pressure usually present with optic neuritis (13, 21, 24), meningoencephalitis (13, 14, 25–27), or acute disseminated encephalomyelitis (ADEM) (28). Two recent retrospective studies found that 21% of patients with positive MOG-antibodies and available CSF opening pressure measurements had raised intracranial pressure (21, 28). Similarly, two other retrospective studies showed that among patients with MOGAD presenting as meningoencephalitis (6–18%), 28–44% had raised intracranial pressure (14, 25). Nevertheless, our three patients with suggested optic nerve sheath enhancement had normal visual acuity and color vision, with no pain on eye movements, making a diagnosis of optic neuritis very unlikely. They also did not present with fever, seizures, or leptomeningeal enhancement to suggest meningoencephalitis, and had no white matter lesions on MRI suggestive of a demyelinating disorder. Moreover, unlike patients with MOGAD and intracranial hypertension who typically present with CSF pleocytosis and elevated protein (25, 28), our patients all had normal CSF contents.

In summary, optic nerve sheath enhancement may be a rare nonspecific radiologic sign of raised intracranial pressure, likely reflecting venous stasis. Its recognition may provide valuable insights into the pathophysiology of IIH and other disorders of CSF pressure, while first and foremost improving patient care by facilitating the avoidance of optic perineuritis misdiagnosis and iatrogenesis.

## Data Availability

The raw data supporting the conclusions of this article will be made available by the authors to any qualified investigator upon reasonable request.
